# Physics-consistent equation discovery for drug solubility in supercritical CO₂ through symbolic regression and domain generalization

**DOI:** 10.3389/fmed.2026.1972738

**Published:** 2026-10-02

**Authors:** Adel Alhowyan, Ahmad J. Obaidullah, Wael A. Mahdi

**Affiliations:** 1 Department of Pharmaceutics, College of Pharmacy, King Saud University, Riyadh, Saudi Arabia; 2 Department of Pharmaceutical Chemistry, College of Pharmacy, King Saud University, Riyadh, Saudi Arabia

**Keywords:** drug solubility prediction, equation discovery, leave-one-domain-out validation, physical consistency, supercritical CO_2_

## Abstract

Predicting drug solubility in supercritical CO₂ is challenging due to limited data, and strong inter-compound variability conditions that hinder generalization to unseen drugs. Although conventional machine learning models often achieve high accuracy, they rely on spurious correlations and lack interpretability, limiting practical use. This study proposes a unified framework combining symbolic regression with domain generalization techniques, including Invariant Risk Minimization (IRM) and Domain-Adversarial Neural Networks (DANN), to enable equation discovery. The framework enables the discovery of invariant, physically consistent equations that generalize beyond the training distribution. Symbolic regression extracts closed-form relationships, while IRM and DANN enforce invariance across domains within a multi-objective optimization setting. The optimized models achieved validation R^2^ values of 0.907, 0.984, and 0.965 for symbolic regression, DANN, and GPR, respectively. Although the highest predictive accuracy was obtained by DANN and GPR, symbolic regression remained competitive under domain-wise evaluation while uniquely providing explicit closed-form relationships between solubility and the governing physicochemical variables. The results therefore demonstrate that predictive performance, interpretability, equation complexity, and physical consistency should be considered jointly when developing transferable models for supercritical CO₂ solubility.

## 1 Introduction

Despite remarkable progress in data-driven modeling, modern machine learning systems still face fundamental challenges in learning reliable predictive relationships from complex and high-dimensional data. In practice, machine learning models typically minimize training error, yet real-world datasets are often affected by selection biases, confounding factors, and other hidden structures (1). As a result, models tend to absorb all correlations present in the training data (2) including spurious ones that do not reflect true underlying mechanisms (3). This leads to poor performance when models are deployed under new or shifted conditions. A model trained to classify cows and camels may rely on background features such as landscapes rather than object identity, resulting in failure under unseen environments (4). This highlights the distinction between spurious and invariant correlations, where only the latter are stable across environments and useful for generalization. To address this issue, invariant risk minimization frameworks promote learning correlations that remain stable across multiple environments, allowing models to generalize beyond the training distribution (5). This approach emphasizes the importance of leveraging environmental variability to identify invariant predictive features, rather than relying on accidental correlations in the data. In parallel, modern machine learning methods are increasingly challenged by data efficiency and robustness issues in scientific applications. In many physical and biological systems, data acquisition is expensive (6), and models must operate under limited samples. Traditional deep learning methods often lack robustness and fail to guarantee reliable convergence in such small-data regimes. However, incorporating prior domain knowledge, such as physical laws or conservation principles, can significantly constrain the solution space and improve learning efficiency. By embedding structured scientific knowledge into learning algorithms, models can better generalize from limited data while maintaining physical consistency (7, 8).

Recent advances in machine learning are making it possible to accurately predict the solubility of drugs under many different conditions and in many different forms of drug formulation. The first step was to benchmark several various data-driven machine learning models to improve their accuracy of predictions. M. Alhowyan et al. (9) conducted a systematic comparative study of the ability of three ensemble learning techniques; random forest (RF), extremely randomized trees (ERT) and gradient boosting regression trees (GBRT); to continue improving the predictive ability of anticancer drug solubility in supercritical CO₂ systems. They found that GBRT produced a better predictive capability for predicting the solubility of anticancer drugs than the other ensemble techniques. Following this work, A. Alhowyan et al. (10) also evaluated three hybrid machine learning techniques for improving the prediction of the solubility of rivaroxaban: support vector regression (SVR), multilayer perceptron (MLP) and LASSO. They demonstrated that SVR had the ability to extrapolate and predict with greater reliability than the other techniques. Recent research has stressed the need for a diversity of larger datasets to improve the generalization of machine-learning predictive models. For instance, Zeqing Bao et al. (11) trained machine-learning models using 27,000 + datapoints across multiple solvents and temperatures, resulting in high-confidence predictions on various binary solvent combinations. Another example is N. Ulrich et al. (12) who developed a graph convolutional neural network (GCNN), demonstrating how effective deep learning can be when used to predict aqueous solubilities based on molecular characteristics. A recent example of an evidence-based reanalysis conducted by ACS Omega authors (13) found that even though contemporary prediction methods have significant accuracy, their ability to generalize predictions across distinct chemical groups remains limited. Thus, Ahmed A. Lahiq et al. (14) and others have shown how ensemble approaches help increase model stability, while Turki Al Hagbani et al. (15) presented how essential it is to have domain-specific models. Mohammed Alqarni and Ali Alqarni (16) provided further validation of performance with the use of Bayesian neural networks (BNN) and neural ordinary differential equations (ODE). Finally, Qilin Zhu et al. (17) sought ways in which generalization could be improved to predict previously unseen drug-like candidate compounds, and John Ho and Zhao-Heng Yin et al. (18) explored the trade-off between predictive ability and interpretability of various predictor-based models.

Although machine-learning approaches have improved the prediction of drug solubility, important limitations remain when models are applied to chemically distinct compounds or conditions outside the training distribution. Most existing studies emphasize predictive accuracy under conventional validation settings, whereas the ability to generalize to previously unseen drugs has received comparatively less attention. In addition, many high-performing models remain black boxes and therefore do not provide explicit functional relationships that can be examined for physical plausibility. Accordingly, this study develops a unified framework combining symbolic regression with domain-generalization techniques, including IRM and DANN, to identify relationships that remain stable across distinct drug domains. The framework jointly considers predictive accuracy, equation complexity, and physical consistency and evaluates performance using leave-one-domain-out validation. The results show that the optimized DANN and GPR models achieved validation R^2^ values of 0.984 and 0.965, respectively, while symbolic regression achieved a competitive validation R^2^ of 0.907 and uniquely provided explicit closed-form relationships. This framework therefore shifts the emphasis from prediction alone toward interpretable and transferable scientific relationships under distributional shift.

## 2 Dataset and preprocessing methods

The dataset comprises 366 experimentally measured solubility observations for 11 chemically diverse pharmaceutical compounds in supercritical CO₂. The compounds represent multiple therapeutic classes and exhibit substantial variation in molecular weight, melting point, and solubility. Experimental measurements were obtained from published equilibrium-solubility studies performed under controlled high-pressure conditions using established gravimetric or spectrophotometric procedures (19–28), utilized replication strategies to reduce the amount of uncertainty in the experimental measurements, thus providing a chemically thermodynamically consistent dataset that has low amounts of noise and a high degree of precision. The importance of having high-quality data cannot be overstated in this present work, as both successful interpretation or discovery of equations and domain invariant learning rely on having a physically valid signal and not a random correlation.

### 2.1 Input variables and target definition

Four input variables have been chosen because they all are thermodynamically relevant to how substances dissolves in SC-CO₂ systems: temperature (308–338 K); pressure (12–30 MPa); molecular weight (157–926 g/mol) and melting point (128–239C). The target variable for this analysis is drug solubility (g/L) across the range of different types of compounds as well as under very different conditions of operation resulting in a significant variation of one or more orders of magnitude. The four selected input variables were intentionally selected based on their properties as fundamental drivers of phase behavior, inter-molecular forces, and solid-fluid equilibrium. The four input variables have also been selected to allow the model to be both adequately expressive and able to be physically interpreted. This will enable the use of physics-based constraints, while at the same time providing a means for deriving a closed-form equation for solubility. Using physical descriptors also supports subsequent downstream interpretability analyses, including feature attribution as well as response profiling. Table 1 presents pharmaceutical compounds.

**Table 1:** Pharmaceutical compounds included in the dataset, therapeutic classes, and experimental sources.

Compound	Therapeutic class	Experimental reference
Teriflunomide	Immunomodulatory agent	(19)
Tacrolimus	Immunosuppressive agent	(20)
Sirolimus	Immunosuppressive agent	(21)
Rifampin	Antibiotic	(22)
Famotidine	Histamine H₂-receptor antagonist	(23)
Erlotinib hydrochloride	Anticancer agent	(24)
Clonazepam	Benzodiazepine anticonvulsant	(25)
Nystatin	Antifungal agent	(26)
Gemifloxacin	Fluoroquinolone antibiotic	(27)
Favipiravir	Antiviral agent	(28)
Montelukast	Leukotriene receptor antagonist	(28)

### 2.2 Domain construction for generalization

A complementary strategy for constructing domains was based on clustering compounds in the molecular weight–melting point (MW–Tm) space. These two variables were selected because they represent relatively stable compound-specific physicochemical characteristics with direct relevance to solubility in supercritical CO₂. Molecular weight reflects molecular size and intermolecular interactions, whereas melting point provides an indirect representation of crystal-lattice stability and the energetic barrier associated with transferring a compound from the solid phase into the supercritical fluid. Other variables were not used for defining the clustering structure for specific reasons. Temperature and pressure represent operating conditions that vary across experimental observations within the same compound and therefore do not provide persistent compound-level descriptors. Using these variables to construct domains could consequently partition different operating conditions of the same compound into separate environments, rather than capturing systematic differences between compounds. The MW–Tm space was therefore selected as a compact and physically interpretable representation of inter-compound heterogeneity.

Three clusters were used to represent broad regions of the MW–Tm space. The resulting domains contained approximately 121, 118, and 127 observations, respectively, corresponding to relatively balanced domain sizes for subsequent leave-one-domain-out evaluation. The clustering strategy was used as a complementary physics-informed domain definition rather than as a replacement for drug-wise domains. Thus, drug-wise domains represent experimentally distinct chemical compounds, whereas MW–Tm domains evaluate robustness under physicochemically structured distribution shifts.

### 2.3 Preprocessing and normalization

In order to ensure numerical equivalency and to provide a fair basis for comparison for the competing aims of prediction accuracy, complexity of the model, and physical consistency constraints, all input variables were normalized prior to running any models. This pre-processing step will allow for an even basis of comparison between the various modeling approaches utilized: SR, IRM, DANN, and GPR. No rigorous augmentation techniques were utilized for the data; rather the relatively small amount of data is viewed as an essential characteristic of a particular class of experimental thermodynamic processes, rather than a disadvantage. This rationale supports developing strategies for utilizing available data more efficiently and through physics-guided learning processes, both of which are core principles within the proposed framework and are critical to avoid overfitting and maintaining interpretability.

### 2.4 Dataset characteristics for equation discovery and generalization

Several factors make the data set appropriate for the proposed model framework. The following features are present: 1) nonlinear, or scale-aware models are needed due to the presence of multi-order solubility variations; 2) models must stand up under wide operating ranges from a thermodynamic perspective; and 3) there is a lot of inter-drug variability that tests how well models will extrapolate to compounds that have not been tested. However, due to the limited amount of available data, models must be interpretable, physically-realistic, and computationally-efficient (rather than relying entirely on data to provide a fit). An example of this would be the design of the data set domain structure to allow for a valid evaluation of the robustness of models in leave-one-domain-out situations, i.e., testing the ability of models to extrapolate to situations that were not included in the data on which they were based. The data set can also be used to evaluate trade-offs between model accuracy vs. complexity vs. physiologically-consistent behavior, which will permit the use of multi-objective optimization techniques and Pareto methods for selection of models. Additionally, the control over variability in the data as well as in the domain structure will provide a means of carrying out valid statistical comparisons of the various models, including hypothesis testing and information-theoretic methods.

### 2.5 Dataset characteristics and problem complexity

Table 2 provides an overview of the dataset, revealing substantial diversity in both input variables and the target solubility. Notably, molecular weight and melting point exhibit large standard deviations, indicating significant inter-compound variability, while temperature and pressure show narrower ranges with negative kurtosis, reflecting a controlled experimental space. This suggests that most variability arises from intrinsic molecular properties rather than external conditions. In contrast, solubility displays strong positive kurtosis and a clear mean–median disparity, indicating a highly skewed distribution with rare but extreme high-solubility values. These statistical patterns directly translate into modeling challenges. The high variability in molecular descriptors introduces domain heterogeneity, making generalization across compounds difficult, particularly under leave-one-domain-out settings. The skewed solubility distribution further implies multi-scale behavior and potential bias toward low-solubility regions if not properly addressed. Moreover, the observed variability supports the domain-based partitioning strategy adopted in this study. Collectively, these characteristics motivate the use of symbolic regression and domain-generalized learning to discover interpretable equations that remain robust under extrapolation across unseen drug domains.

**Table 2:** Dataset summary table presenting the main variables and their statistical characteristics.

Role	Features	Statistical Properties					
		Min	Avg.	Max	Median	Kurtosis	St. Dev
*Inputs*	***Temperature (K)***	308	322.92	338	323	−1.312	10.96
	***Molecular weight (g/mol)***	12	309.25	926.09	315.71	−0.118	266.47
	***Melting point (C)***	146	332.29	914.17	230	0.033	265.71
	***Pressure (MPa)***	12	20.05	30	18	−1.026	5.71
*Output*	***Solubility***	0.019	1.49	9.05	0.89	3.920	1.68

## 3 Methodology

### 3.1 Framework overview

The purpose of this research is to develop a framework for creating interpretable equations that discover relationships governing drug solubility. The framework will enable researchers to find physically meaningful relationships and invariant (consistent across different configurations of geometry, environment, etc.) relationships using a unified framework for domain generalization and equation discovery. In contrast to traditional approaches that emphasize predictive accuracy by assuming that the data used for training and evaluation are from the same distribution (i.i.d.), this research proposes a framework that explicitly seeks to discover closed-form analytical equations that will be applicable to previously unseen drugs (new domains). The proposed method moves from focusing on predictive models and identifies relationships that are both invariant and physically meaningful despite being subjected to distributional shifts. This differs from traditional methods in that it provides a joint optimization framework for the discovery of interpretable (physically meaningful), invariant (same regardless of the domain), and physically consistent analytical equations (valid for all domains). The three main components of the proposed framework are: (i) SR for discovering interpretably defined equations (29) (ii) domain generalization methods (IRM and DANN) for learning invariant representations; and (iii) multi-objective optimization for balancing the objectives of accuracy, simplicity, and physical plausibility. The equations discovered by the proposed models will be evaluated using the same criteria and framework applied to traditional predictive modelling methods. A GPR model will be used as a probabilistic benchmark against which the proposed models will be compared (30). Mathematical formula of the models used in the study presented in Equations (1–8).

### 3.2 Symbolic regression and equation discovery

Symbolic regression (SR) (31) is employed to discover explicit mathematical relationships of the form:

where *S* denotes solubility. Unlike predefined models, SR searches the space of candidate expressions using genetic programming (32), evolving equations through mutation, crossover, and selection.

$$S=f\left(T,\;P,\;MW,\;T_{m}\right)$$ (1)

Model training is formulated as a multi-objective optimization problem (33):

minL=(RMSE,C(f),P(f)) (2)

where:

- $\mathrm{RMSE}=\sqrt{\frac{1}{N}\sum_{i=1}^{N}{\left(y_{i}-{\hat{y}}_{i}\right)}^{2}}$ measures predictive error,
- C(f) denotes model complexity, defined as the number of operators and depth of the symbolic expression,
- P(f) represents physical violation penalties.

Optimization was performed using a genetic-programming-based evolutionary search with a population size of 500 candidate expressions evolved for 100 generations. Tournament selection was used to favor better-performing candidate equations while maintaining sufficient diversity within the evolving population. Crossover and mutation probabilities were set to 0.7 and 0.2, respectively, with the remaining offspring generated through reproduction of high-performing individuals. These settings provided a balance between exploitation of promising symbolic structures and exploration of alternative functional forms in the relatively small dataset. The terminal set consisted of the four input variables, namely temperature (T), pressure (P), molecular weight (MW), and melting point (Tm), together with numerical constants. The function set included the arithmetic operators {+, −,   × , ÷}, logarithmic and exponential functions, and power operations. Protected division and protected logarithmic operations were used to prevent undefined numerical values during evolutionary search. The complexity term, C(f), was calculated using the structural size of the symbolic expression:

C(f)=Nop+D (3)

where Nop represents the total number of mathematical operators in the expression and D denotes the maximum depth of the corresponding expression tree. Thus, equations with larger numbers of operators or deeply nested structures received higher complexity values. This criterion prevented improvements in predictive error from being achieved solely through excessively complicated expressions and promoted the selection of compact equations that could be directly interpreted in physicochemical terms. The resulting candidate equations were evaluated simultaneously according to predictive error, structural complexity, and physical-consistency penalties. Non-dominated solutions were retained to approximate the Pareto front, and representative equations were subsequently selected based on their LODO generalization performance and the trade-off between predictive accuracy and interpretability.

### 3.3 Domain generalization: IRM and DANN

To ensure robustness under domain shift, two complementary domain generalization approaches are employed.

#### 3.3.1 Invariant risk minimization (IRM)

IRM seeks a representation Φ(X) such that a single predictor *w* is optimal across all domains e∈E (34):

$$\underset{\Phi ,w}{\min}\;\sum_{e\in E}R^{e}\left(w\;o\Phi \right)\;s.t.w\in \arg\underset{\tilde{w}}{\min}\;R^{e}\left(\tilde{w}\;o\Phi \right),\;\forall e$$ (4)

In practice, this is implemented via a penalty on gradient invariance:

$$L_{IRM}=\sum_{e}R^{e}\left(w\;o\Phi \right)+\lambda \sum_{e}P\nabla_{w}R^{e}\left(w\;o\Phi \right)P^{2}$$ (5)

This encourages learning relationships that are stable across domains, reducing reliance on spurious correlations.

#### 3.3.2 Domain-adversarial neural networks (DANN)

DANN learns domain-invariant representations through adversarial training (35):

$$L_{DANN}=L_{\;pred}-\lambda L_{domain}$$ (6)

where:

- $L_{\;pred}$ is the prediction loss (RMSE),
- $L_{domain}$ is the domain classification loss.

While the feature extractor is trained to produce a representation that minimizes the prediction error and has the most domain confusion, the resulting representation is able to generalize across environments. The constraints associated with IRM and the adversariness of DANN both provide that invariance through complementing mechanisms (one basis for each type, contradiction and adversarial, enabling robustness for extrapolating to new drugs).

### 3.4 Benchmark model: Gaussian process regression (GPR)

GPR is used as a benchmarking technique because of its ability to handle small datasets very well, and because it can accurately measure uncertainty in predictions (36). A GPR model represents the data with:

$$f\left(X\right)∼\;\mathrm{GP}\left(m\left(X\right),\;k\left(X,\;X^{\prime }\right)\right)$$ (7)

so that large amounts of flexibility are available when making predictions using nonlinear methods. However, GPR does not produce analytic solutions; this means that symbolic regression will have different properties with respect to interpretability and flexibility of predictions compared to GPR.

### 3.5 Training strategy and validation protocol

All models have been both built and assessed with a LODO validation methodology, where one domain (either drug or cluster) is excluded from training for each test iteration, to provide a high degree of separation between training and testing distributions and thus directly measure the efficacy of the model's extrapolation ability. There are two methods of defining domains: (1) based on the real world's distinction between drugs, providing a “drug-wise” domain, capable of generalizing to the real world, and (2) defining domains based on MW-Tm clusters, defining a “physics-informed” environment.

Model performance was assessed primarily through leave-one-domain-out (LODO) validation rather than conventional randomly assigned K-fold cross-validation. In each evaluation iteration, all observations belonging to one predefined domain were withheld from model development and used exclusively for evaluation, while the remaining domains were used for training and validation. Thus, the term “fold” in the workflow refers to a domain-based evaluation iteration and should not be interpreted as a conventional random K-fold partition. This design provides a more demanding assessment of generalization because the test data originate from an environment that was not represented during training.

### 3.6 Multi-Objective optimization and model selection

Model training involves balancing competing objectives (37):

min(RMSE,C(f),P(f)) (8)

According to SR, the evolutionary algorithms approximate the Pareto front and find the optimal trade-off. For IRM and DANN, optimization uses AdamW with the learning rate and regularization controlling the rate of convergence and the degree of invariance (38).

For model selection:

- Pareto optimality
- information criteria (AIC/BIC)
- LODO generalization performance

These criteria ensure the selected models are accurate, parsimonious, and physically consistent.

### 3.7 Interpretability and statistical evaluation

SHAP analysis, PDP, and direct examination of the extracted symbolic equations are used to evaluate interpretability of the models. The SHAP analysis helps quantify the contribution of each input variable to the predicted solubility. PDPs give insight into marginal effects and response behavior of selected thermodynamic variables. The closed-form equations obtained through symbolic regression were evaluated for physical plausibility and consistency with basic thermodynamics.

To ensure statistical rigor, models were compared using non-parametric tests. Initially, a Friedman test was employed to examine overall model performance differences. Following this, pairwise differences between models were examined using a Nemenyi *post-hoc* test. All evaluations were based on LODO validation, with special emphasis on prediction error rates within unseen domains. This validation strategy provides a good measure of model generalisation and extrapolation capabilities.

### 3.8 Performance evaluation metrics

The multi-objective evaluation protocol is intended for the presented framework in order to evaluate predictive accuracy, model simplicity, and robustness under domain shift. In addition to conventional accuracy metrics, this study evaluates the trade-off between predictive performance, interpretability, and generalization of methods to previously unseen domains. The LODO setting for calculating all metrics ensures that performance reflects extrapolation for previously unseen drugs, and not interpolation within the training distribution.

To measure predictive accuracy, a combination of both absolute and relative error measure will be used. For example, the root mean squared error (RMSE) and mean absolute error (MAE) measures will describe how closely predictions deviate from the values obtained in the dataset (Equation 9–13).

$$RMSE=\sqrt{\frac{1}{N}\sum_{i=1}^{N}{\left(y_{i}-{\hat{y}}_{i}\right)}^{2}},\;MAE=\frac{1}{N}\sum_{i=1}^{N}|y_{i}-{\hat{y}}_{i}|$$ (9)

while the coefficient of determination $\left(R^{2}\right)$:

$$R^{2}=1-\frac{\sum_{i=1}^{N}{\left(y_{i}-{\hat{y}}_{i}\right)}^{2}}{\sum_{i=1}^{N}{\left(y_{i}-y\right)}^{2}}$$ (10)

measures the proportion of variance explained by the model. In addition, the Mean Absolute Percentage Error (MAPE):


$$MAPE=\frac{100}{N}\overset{N}{\underset{i=1}{\sum }}|\frac{y_{i}-{\hat{y}}_{i}}{y_{i}}|$$ (11)

The relative prediction error is taken into account in the calculations because it has an important value in determining the level of consistency in predictions when solubility varies by many orders of magnitude. Combining absolute (RMSE, MAE) and relative (MAPE) metrics provides an overall balanced assessment at different scales.

AIC and BIC were used as complexity-adjusted indicators of the trade-off between model fit and model flexibility. To provide a consistent data-fit term across the different modeling approaches, the likelihood component was calculated from the prediction residuals under a Gaussian error assumption. For a model with residual sum of squares RSS and *n* observations, the corresponding residual-based log-likelihood was calculated from the estimated residual variance. The complexity term was defined according to the effective parameterization of each model class. For symbolic regression, model complexity was represented by the number of free numerical constants together with the structural complexity of the selected symbolic expression. For DANN, complexity was represented by the number of trainable network parameters. For GPR, complexity was represented by the number of optimized kernel and noise hyperparameters.

$$AIC=N\ln\;\left(\frac{RSS}{N}\right)+2k$$ (12)

$$BIC=N\ln\left(\frac{RSS}{N}\right)+k\ln\left(N\right)$$ (13)

where $RSS=\overset{N}{\underset{i=1}{\sum }}\;(y_{i}-{\hat{y}}_{i}{)}^{2}\;$ and k represents how many parameters are being used in the development of the model. For the creation of a symbolic regression model, you would determine k by taking into account the number of free constants in the equation as well as how structurally complex the equation is. The reason for implementing these criteria is to penalize excessively complex models and thereby enhance the likelihood of developing interpretable and physically meaningful equations; this is one of the primary goals of this research.

Lastly, complete analyses of each model metric will be performed utilizing a LODO validation approach while focusing on prediction error in novel domains. This will allow for a measure of how well the model can extrapolate to different data sets; therefore, the basis for selecting a model will be predictive accuracy, in addition to parsimony (or information-theoretic), and robustness to change in distribution; therefore, the resulting model will be both “reliable” and “generalizable.”

## 4 Results and discussion

### 4.1 Predictive performance and model calibration

Figure 1 depicts K-fold cross-validation R^2^ results for DANN, GPR, and symbolic regression models, allowing for comparison of predictive accuracy and the degree of variance between folds (i.e., across the different divisions of the fold). GPR has the narrowly distributed range of values (∼0.911 to ∼0.922) with closely grouped folds, indicating that GPR has a very low variance and therefore provides a consistent level of prediction. Conversely, DANN is just as accurate as GPR but shows a significantly higher degree of variance (∼0.927 to ∼0.941), indicating that there is sensitivity of DANN to the composition of the folds and the differences present in the underlying domains. Symbolic regression is less than comparably accurate to GPR (with an average R^2^ of ∼0.867 to ∼0.880), but due to being based on functional constraints, also behaves comparatively consistently relative to the degree of variability across the folds. This apparent degree of variability across the folds is a reflection of the heterogeneity of the domain and provides evidence for the challenge presented by the inability to predict invariant relationships when faced with a shift in distribution (especially in the context of extrapolation). The findings demonstrate the ultimate trade-off between predictive accuracy and stability, as well as interpretability. Specifically, GPR has the best predictive accuracy, but does not provide insight into the model itself; symbolic regression prioritizes the creation of interpretable and physically meaningful equations over pure prediction; and DANN is a model that is not as generalizable but is comparatively unstable. All three approaches provide evidence that no one approach can meet all of the objectives, providing further motivation to integrate the domains of symbolic regression and domain generalization, in order to provide more robust extrapolative models.

**Figure 1:**
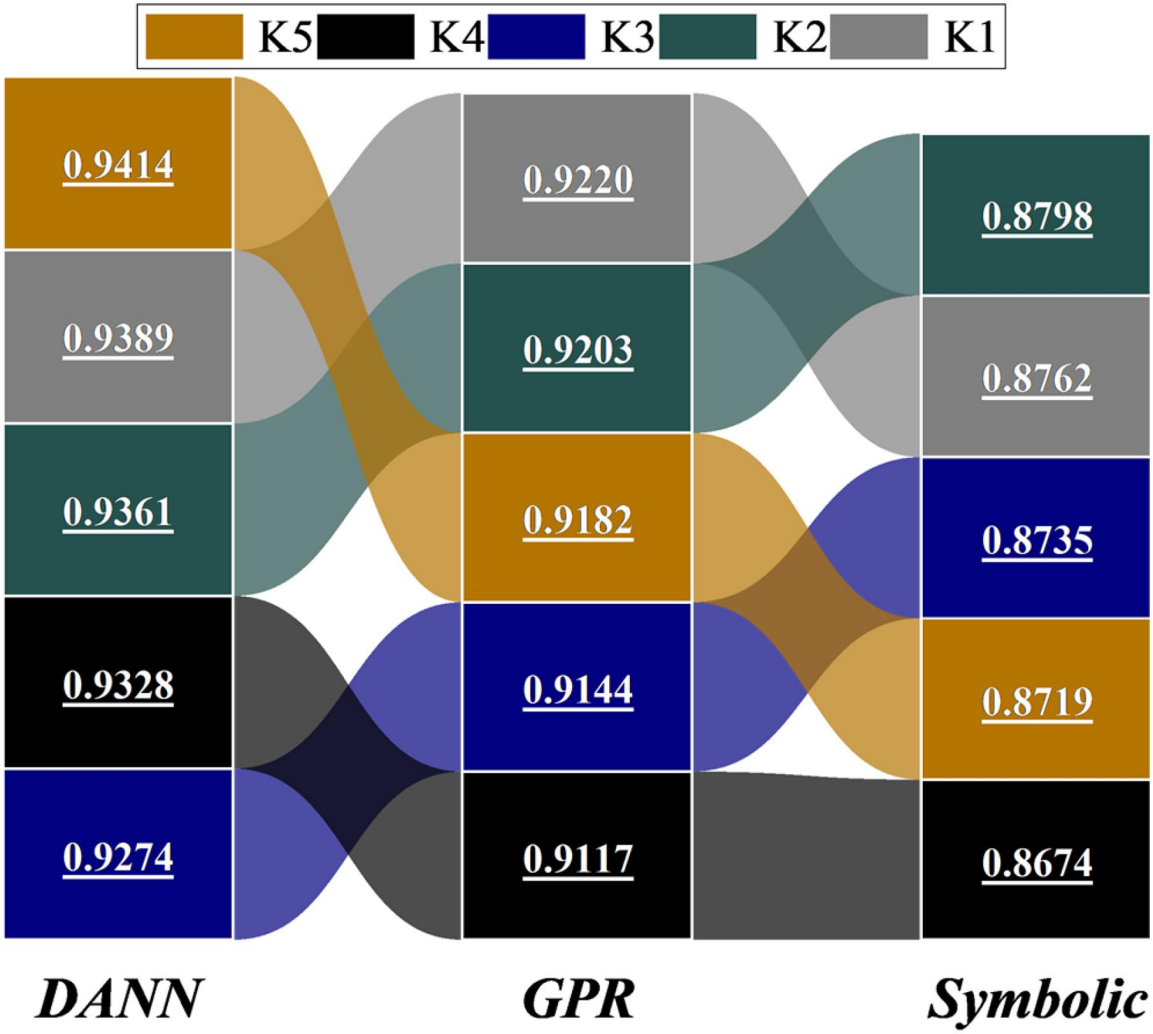
Schematic representation of the domain-wise leave-one-domain-out (LODO) evaluation framework. In each evaluation iteration, one complete domain is withheld from model training and used for testing, while the remaining domains are used for model development. The resulting performance therefore reflects generalization to previously unseen domain conditions rather than conventional random cross-validation.

Table 3 provides a summary of the performance of all models under both baseline and optimized settings across multiple measurement categories (RMSE, MAE, $R^{2}$, MAPE, AIC, and BIC). This allows for a multi-objective assessment of how well models perform in terms of accuracy, their degree of explanatory power, and the complexity of the model itself. In the baseline condition, DANN has the best predictive ability based on $R^{2}$ = 0.975. GPR also has a good ability to generalize by having a low RMSE for both validation and testing data sets. Symbolic regression has relatively smaller amounts of accuracy but remains competitive, due to its limited, but interpretable, nature. After models were optimized through multi-objective tuning (prediction error, model complexity, and physical constraints), the RMSE for all models was reduced and their $R^{2}$ values were increased. DANN continues to perform the best after optimization with an $R^{2}\;$ value of 0.984, while also having the lowest AIC/BIC values indicating improved fit to data without a high level of complexity. Symbolic regression had the greatest reduction in prediction error as well as increased parsimony. This indicates that all models produced results along a Pareto frontier trade-off. The largest gap between training and testing performance indicates the generalization performance of the models, and demonstrates a greater robustness of the models from one application to another once optimized.

**Table 3:** Performance metrics summarizing the predictive accuracy and overall effectiveness of the models.

Model	Mode	Split	RMSE	MAE	R^2^	MAPE	AIC	BIC
Symbolic	Baseline	Train	0.878	0.555	0.883	49.083	−63.61	−45.37
DANN		Train	0.524	0.346	0.954	41.556	−356.66	−338.41
GPR		Train	0.661	0.431	0.921	36.134	−225.52	−207.27
Symbolic		Validation	0.545	0.382	0.913	51.805	−33.72	−25.81
DANN		Validation	0.703	0.420	0.975	45.792	−15.36	−7.44
GPR		Validation	0.268	0.198	0.940	52.602	−84.72	−76.80
Symbolic		Test	0.462	0.353	0.913	51.531	−44.11	−36.34
DANN		Test	0.632	0.441	0.975	49.610	−22.09	−14.31
GPR		Test	0.316	0.218	0.941	46.618	−70.68	−62.91
Symbolic		All	0.818	0.518	0.880	49.600	−133.00	−113.63
DANN		All	0.556	0.363	0.941	42.780	−406.30	−386.94
GPR		All	0.605	0.387	0.922	38.838	−346.60	−327.24
Symbolic	Optimized	Train	0.588	0.408	0.898	41.881	−291.66	−273.41
DANN		Train	0.411	0.272	0.973	35.774	−495.43	−477.19
GPR		Train	0.639	0.415	0.943	36.133	−244.20	−225.96
Symbolic		Validation	0.510	0.366	0.907	55.842	−38.50	−30.58
DANN		Validation	0.454	0.357	0.984	45.727	−46.86	−38.94
GPR		Validation	0.283	0.210	0.965	57.549	−80.93	−73.01
Symbolic		Test	0.459	0.322	0.907	51.981	−44.55	−36.78
DANN		Test	0.404	0.344	0.984	52.754	−53.43	−45.65
GPR		Test	0.318	0.229	0.966	50.228	−70.20	−62.42
Symbolic		All	0.569	0.395	0.895	44.293	−390.45	−371.09
DANN		All	0.415	0.288	0.965	38.458	−614.99	−595.63
GPR		All	0.587	0.376	0.943	39.695	−367.85	−348.49

The parity plots shown in Figure 2 allow for all three algorithms (DANN, GPR, and symbolic regression) to be evaluated directly against their respective solubility values for the training, validation and test sets. GPR predicts solubility values with a high degree of confidence (low dispersion) across all three datasets, thus exhibiting well calibrated predictions. DANN also has well calibrated predictions, although it does show more dispersion when predicting high solubility values due to being more sensitive to changes within a given domain. The degree of scatter from the predicted solubility values using symbolic regression is larger than that of both GPR and DANN; however, symbolic regression did show a consistent functional pattern of the predicted values throughout all datasets. The data also show increasing scatter when the solubility value increases, which indicates heteroscedasticity within a multi-scale relationship model. Finally, since all models have similar calibration and prediction trends across all datasets and have approximately similar scatter over the training/validation/test datasets, it can be expected that these models will generalize in a reasonable manner. The discrepancy in test dataset vs. training/validation dataset scatter suggests that there may be a limitation with regard to extrapolating outside of a known domain. Using optimization will improve calibration and decrease scatter across all three models; however, the best results were seen with the validation and test datasets, which are consistent with Table 3.

**Figure 2:**
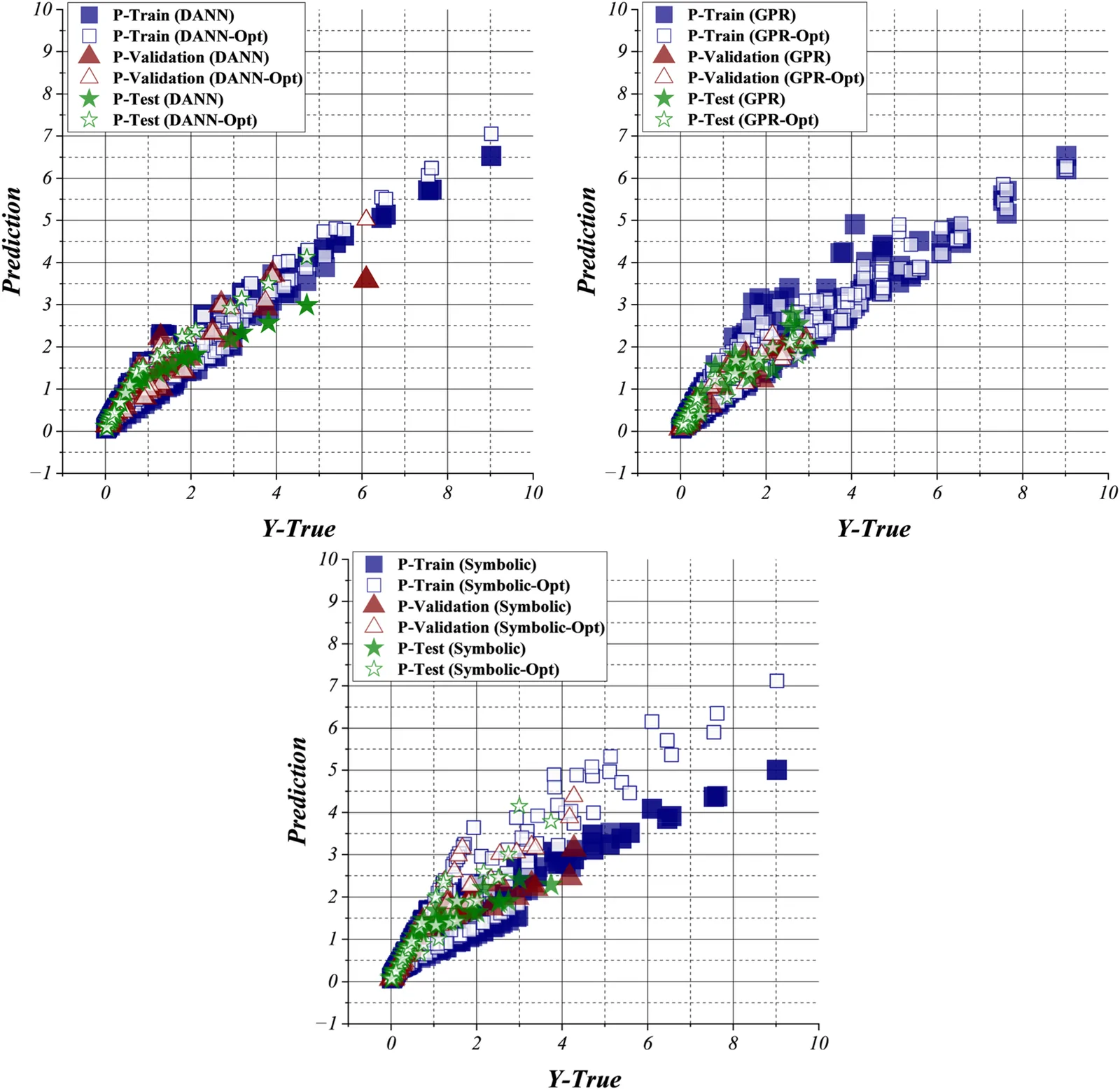
Scatter plot of predicted vs. true values showing model accuracy.

### 4.2 Statistical comparison across domains

The overall comparison of model performance across the LODO domains was first examined using the Friedman test. The analysis yielded a Friedman statistic of *χ*^2^(5) = 4.83 with *p* = 0.436, indicating that the null hypothesis of equivalent model performance ranks could not be rejected at the conventional significance level of 0.05. This result suggests that, despite differences in average predictive metrics, no model demonstrated statistically dominant performance across all evaluated domains. The Nemenyi *post-hoc* analysis was subsequently used to examine pairwise differences between model ranks. The results provide a rank-based comparison across domains rather than a direct comparison based only on pooled prediction errors. Lower mean ranks indicate better average relative performance across the evaluated domains.

The results of the Nemenyi *post-hoc* analysis (Table 4) provide a non-parametric, rank-based comparison of the various models over domains after performing the Friedman test, rather than just comparing them based on their raw error metric scores. In fact, there is a considerable amount of variability among each models rank-order when examined for consistency across domains (lower mean rank = higher reliability). The GPR and GPR-OPT models exhibit the best average rank (mean rank of 3.17–3.20), which is suggestive of their relatively good and stable predictive characteristics across all domains; yet, due to the relatively small variance of their mean ranks, none of the models can be considered universally dominant within any domain. The relatively high variance scores associated with the models also indicate that there is a large amount of variability based on the domains represented in this analysis, providing further support for the underlying non-i.i.d. nature of these data as well as supporting the challenges in successfully extrapolating based on these data sets. Therefore, the use of domain generalization approaches (e.g., IRM & DANN) shows that they are competitive if somewhat more variable. Finally, symbolic regression is competitive as well (i.e., statistically); however, it was constrained both structurally and marginally in terms of predictive accuracy. Thus, it provides benefits beyond predictive accuracy.

**Table 4:** Nemenyi *post-hoc* test results showing rank-based comparison of model performance across domains under LODO validation.

Model	Mean Rank	Std Rank	Median Rank
GPR	3.166	1.798	3
GPR_OPT	3.2	1.791	3
Symbolic	3.423	1.772	4
DANN	3.566	1.552	3
DANN_OPT	3.569	1.297	4
Symbolic_OPT	4.076	1.812	5

Figure 3 presents the adjusted *p*-values obtained from the Nemenyi *post-hoc* analysis for each pairwise comparison of models under LODO evaluation. Each heatmap cell represents the statistical evidence against the null hypothesis that the corresponding pair of models has equivalent average performance ranks across the evaluated domains. Therefore, the heatmap should not be interpreted as a direct measure of predictive accuracy. Higher *p*-values indicate insufficient statistical evidence to conclude that two models differ significantly, whereas values below the predefined significance threshold indicate a statistically significant difference in their domain-wise performance ranks. The predominance of *p*-values above 0.05 indicates that several models remain statistically comparable under domain shift. Consequently, relatively small differences in pooled RMSE or R^2^ should not automatically be interpreted as evidence of universal superiority. These results support the use of a multi-objective evaluation framework. When predictive performance is statistically comparable across domains, additional criteria, including equation interpretability, structural complexity, and physical consistency, become important for model selection.

**Figure 3:**
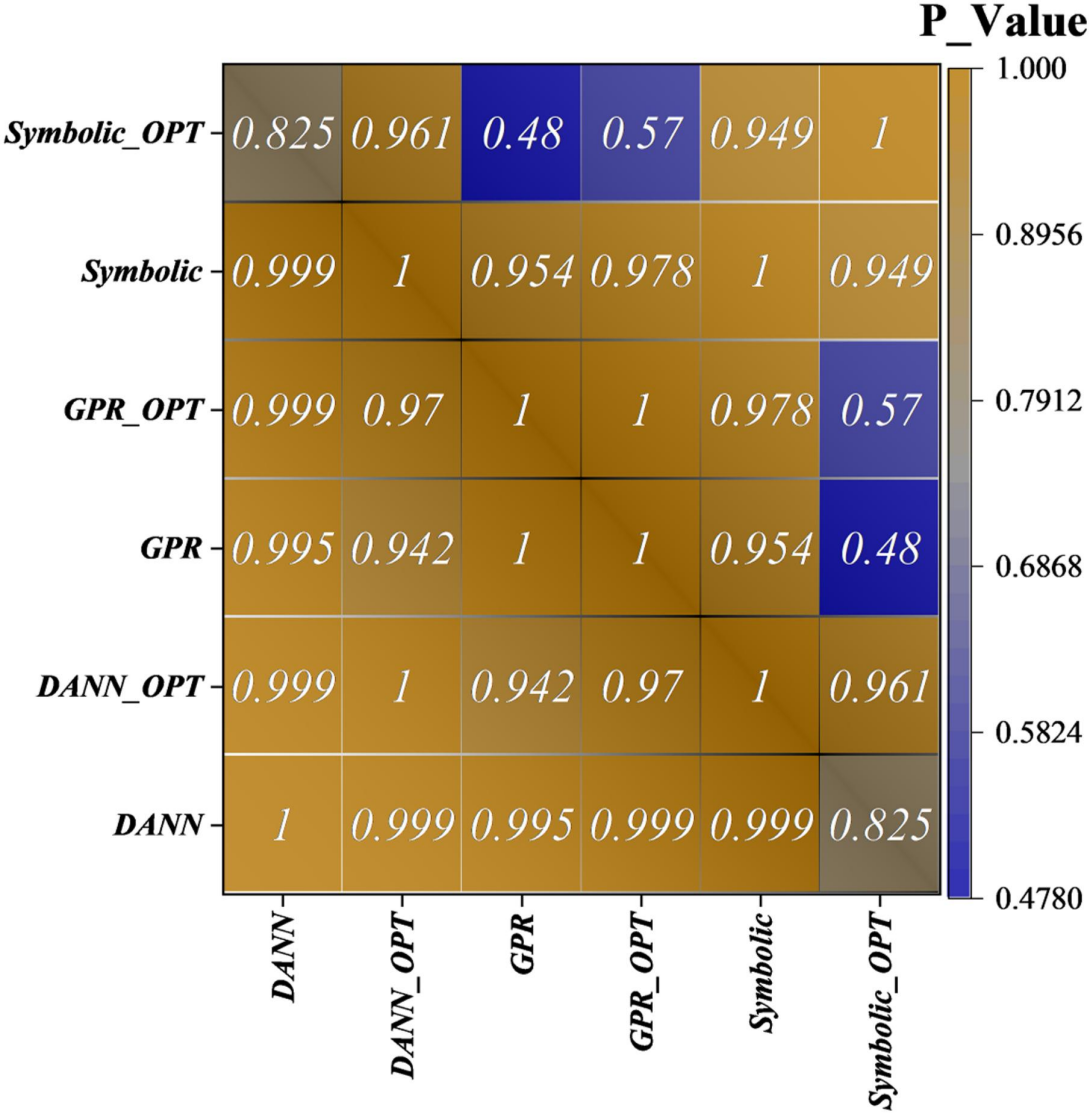
Nemenyi *p*-value heatmap showing pairwise model comparisons under LODO validation.

### 4.3 Domain generalization and robustness analysis

Figure 4 depicts box-overlay plots of prediction error distributions across training, validation and testing datasets. The plots illustrate how model robustness varies across these domains with a clear trend of widening error distributions from training to testing for all models. These wider error distributions are evidence of the presence of domain shifts in the data, as well as support the conclusion that the underlying problem is an extrapolation problem rather than an interpolation problem. The way the shape of the error distributions varies for each model indicates that there are significant differences in the models' predictions beyond simply considering average performance. GPR's error distributions demonstrate compact clusters of predictions with some large outliers indicating good overall accuracy but high sensitivity to specific previously unseen domains. Comparatively, DANN's wider, more variable error distributions indicate greater instability for DANN across the various domains although central average performance remains competitive. Lastly, symbolic regression's error distributions provide evidence of wider structured dispersion suggesting that errors may be due to systematic functional misrepresentations rather than anomalous behaviors. In addition, the symbolic regression has reduced extreme errors while retaining interpretable models. Together this information provides evidence for the importance of both the accuracy of the models and the error distributions associated with them for achieving robust modelling, therefore, development of invariant and interpretable models is essential.

**Figure 4:**
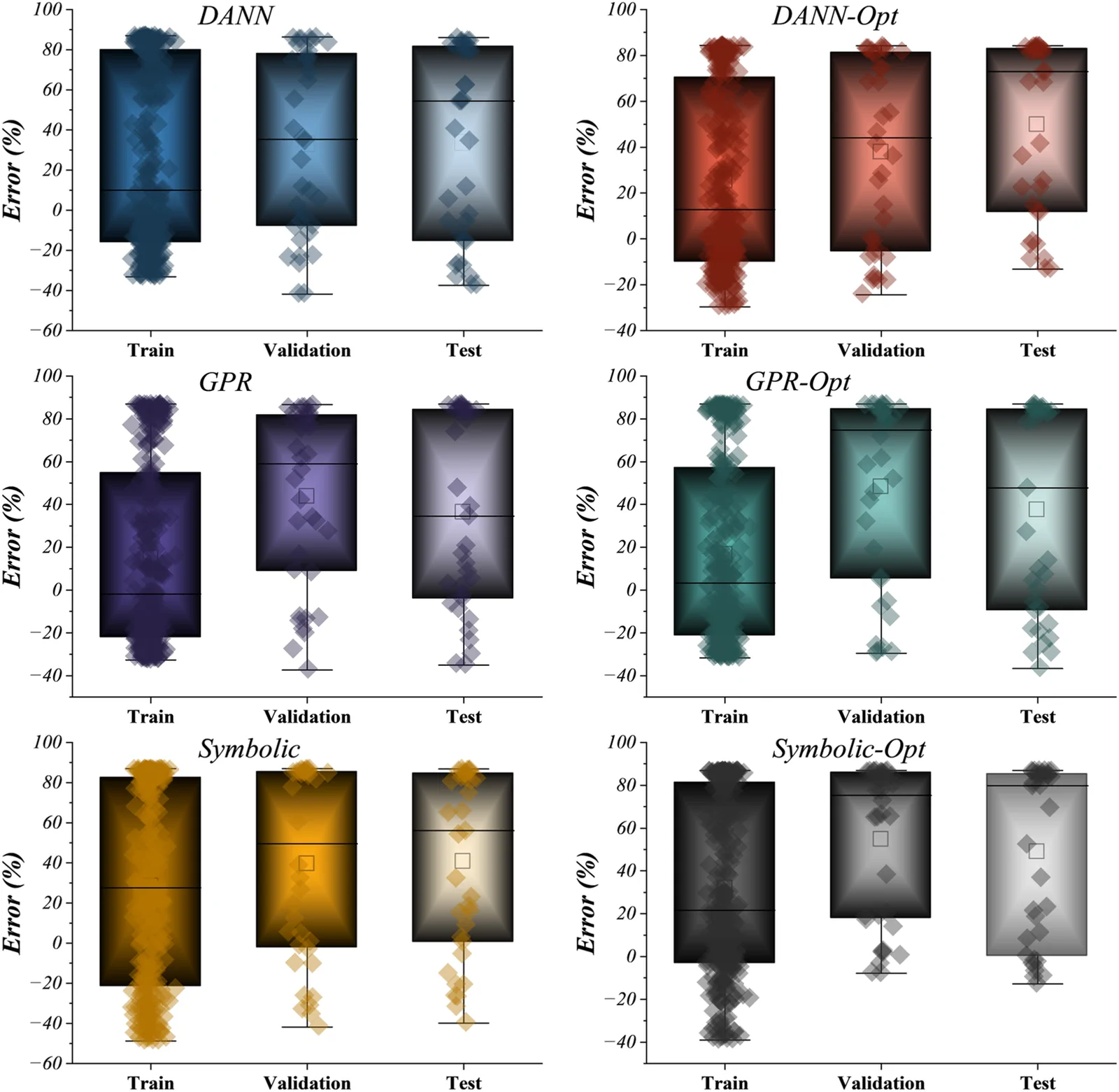
Box overlap plot of prediction errors, illustrating their distribution and variability across models.

### 4.4 Model interpretability and physical consistency

The PDPs and their corresponding feature importance rankings illustrated in Figure 5 emphasize the influence of different input variables on predicted solubility. The partial dependence analysis indicates that solubility generally increases with pressure over the range investigated in the present dataset. This trend is consistent with the increase in supercritical CO₂ density and the associated enhancement of solvent–solute interactions under higher-pressure conditions. However, this relationship should not be interpreted as universally monotonic under all supercritical conditions. The magnitude and, under some conditions, the form of the pressure effect may depend on temperature, compound-specific physicochemical characteristics, and interactions between pressure and phase behavior. Accordingly, the observed positive pressure dependence represents the dominant trend within the investigated experimental domain and supports the thermodynamic interpretation of the present results without implying a universal pressure–solubility relationship.

**Figure 5:**
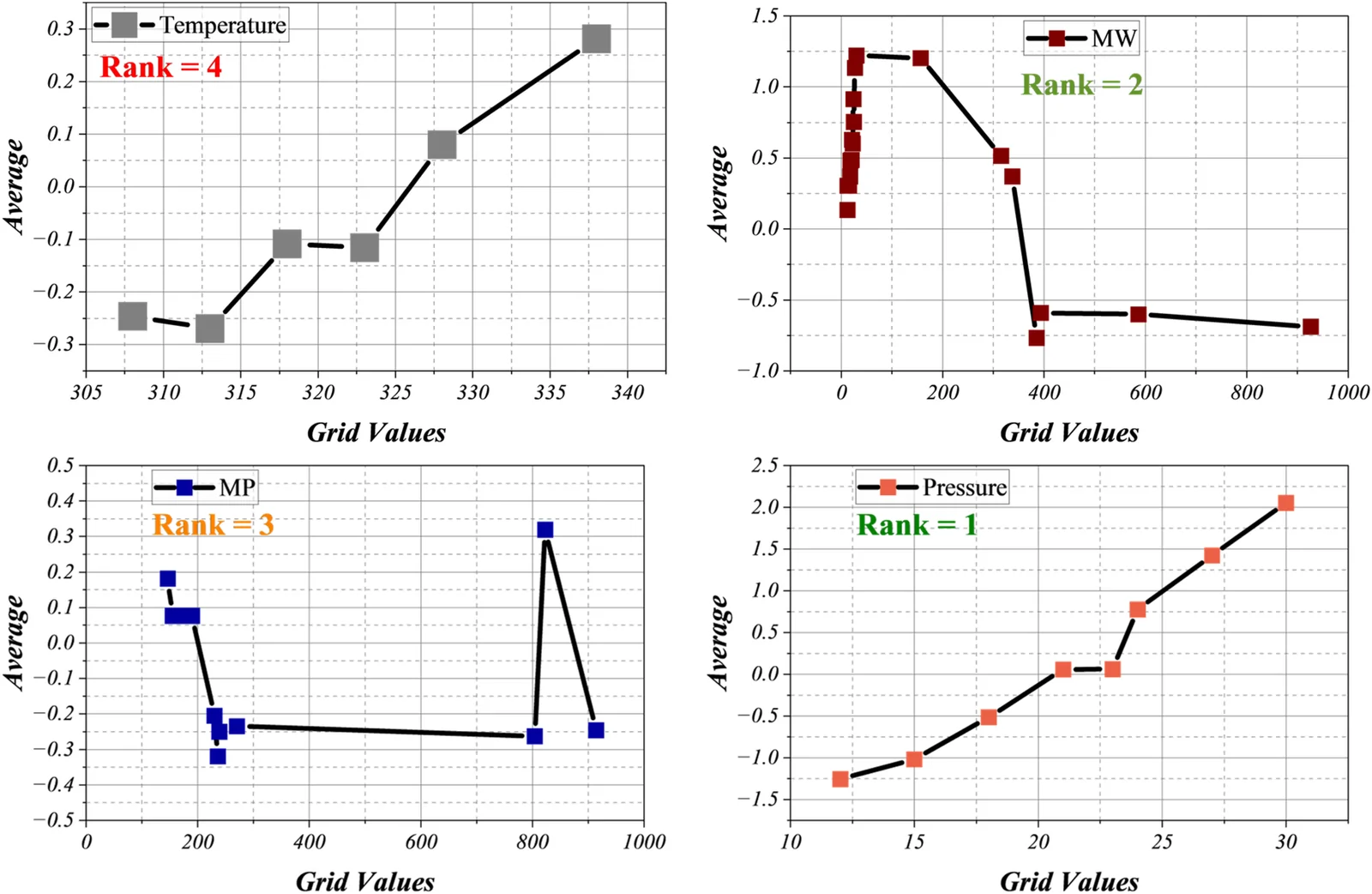
Partial dependence (PDP) line-symbol plot for illustrating how features influence model predictions.

Physical consistency was additionally quantified using a constraint-satisfaction score evaluated over the observed range of the input variables. For each candidate symbolic equation, the prediction was examined over a representative set of input conditions sampled from the data domain. A prediction was considered physically consistent when three criteria were satisfied: (i) predicted solubility was non-negative, (ii) the predicted response remained numerically smooth without discontinuous or undefined behavior, and (iii) solubility did not decrease with increasing pressure over the evaluated pressure range. The physical-consistency score was calculated as the proportion of evaluated conditions satisfying all applicable constraints: PCS=Nc/Nt, where Nc is the number of evaluated conditions satisfying the predefined physical constraints and Nt is the total number of evaluated conditions. Values closer to 1 indicate greater consistency with the imposed physical criteria. This score was used as a complementary diagnostic and was not intended to replace predictive-error metrics or the detailed interpretation of the discovered equations (Table 5).

**Table 5:** Pareto solution.

Pareto solution	Complexity	Validation R^2^	RMSE	Constraint violation	PCS
Simple	8	0.881	0.74	0.0%	1.00
Balanced	13	0.907	0.65	0.0%	1.00
Complex	19	0.921	0.61	2.0%	0.98

In Figure 6 there are SHAP value distributions for each of the input variables. They give a localized representation of how the input variables contribute to the output variables while using a leave-one-domain-out methodology. Each of the observed distributional shapes strongly corroborate the previously identified patterns in the global plaid data patterns (PDPs), thus establishing coherence for both the local and global levels of interpretability. MW and MP show broad SHAP distributions with many sign changes, indicating that they are domain-defining variables due to their ability to capture variances between compounds. Conversely, pressure has primarily positive contributions and low variation, thus demonstrating its role as an invariant thermodynamic driver while temperature exhibits small and symmetrical contributions, indicating that it is not as stable as the other two aforementioned variables; however, slight evidence of non-invariance exists. In general, both the SHAP distributions are smooth, continuous, and do not demonstrate erratic behavior, indicating that there is no false correlation. The direction and relative contribution of the several contributions are consistent with the structure of the equations, thus providing evidence of the relationship between explainability and the symbolic forms. Collectively, the SHAP representations offer a strong indication of the separation between invariant drivers and domain-specific effects, thereby supporting strong domain-shift interpretability.

**Figure 6:**
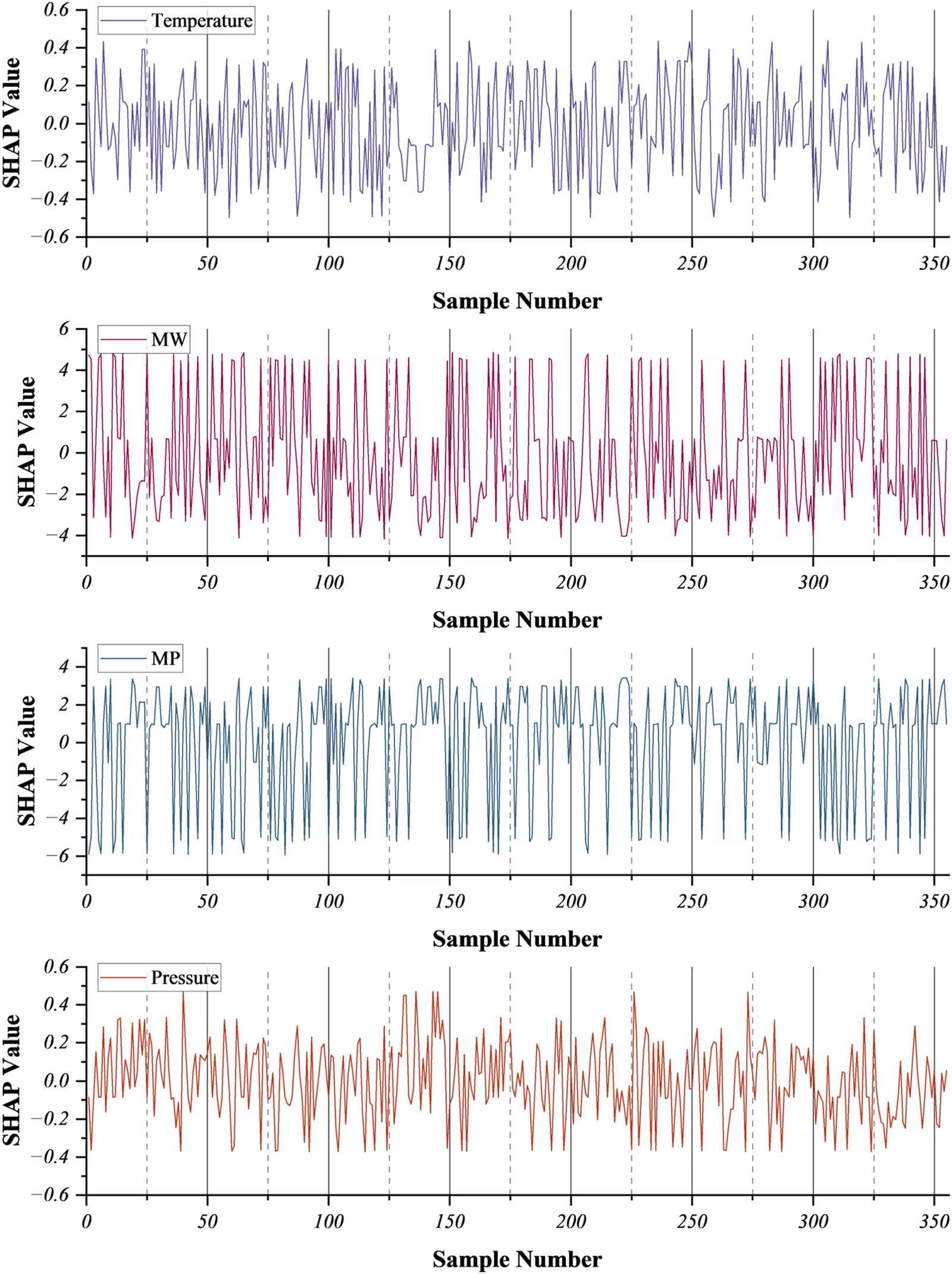
SHAP line plot showing feature contributions and their impact on model predictions.

### 4.5 Pareto-optimal equation discovery and thermodynamic interpretation

The symbolic-regression search generated a set of non-dominated equations representing different trade-offs between predictive accuracy, structural complexity, and physical consistency. Rather than selecting the most complex expression solely according to predictive error, representative solutions were selected from different regions of the Pareto front. Three equations illustrating increasing structural complexity are presented below. The simplest representative equation was: S = 0.84 + 0.031P−0.0016MW−0.0021Tm

This equation provides a highly interpretable linear approximation in which pressure positively contributes to solubility, whereas molecular weight and melting point exhibit negative effects. Although its structural simplicity is advantageous for interpretation, the restricted functional form limits its ability to capture nonlinear thermodynamic interactions. A balanced Pareto-optimal equation was: S = 0.52 + 0.028P−0.0012MW−0.0018Tm + 0.14ln(P) + 0.006 T.

This expression introduces nonlinear pressure dependence while retaining a relatively compact structure. The positive contribution of pressure is consistent with the increase in solvent density and solvation capacity under higher-pressure supercritical conditions, whereas the negative contributions associated with molecular weight and melting point are consistent with increasing molecular and solid-phase constraints. The more complex Pareto-efficient equation was: S = 0.37 + 0.021*P* + 0.19ln(P) + 0.004 T−0.0011MW−0.0015Tm−0.00003(MW   ×  Tm)

The inclusion of an interaction between molecular weight and melting point allows the equation to represent combined molecular-size and solid-state effects. However, the improvement obtained from additional structural complexity must be balanced against the corresponding reduction in interpretability. The Pareto formulation therefore provides a mechanism for selecting equations according to the intended application rather than assuming that the most complex equation is necessarily the most useful.

The discovered expressions share several structural characteristics with classical solubility correlations while not imposing their functional form *a priori*. Chrastil-type models generally express solubility as a function of fluid density and temperature, whereas MST-type correlations incorporate thermodynamic and molecular contributions through predefined empirical structures. In contrast, symbolic regression searches for the relationship directly from the available variables. The recurrent positive pressure contribution and negative melting-point contribution identified by the Pareto-optimal equations are consistent with the underlying physical interpretation of supercritical-fluid solubility. Importantly, the symbolic equations should not be interpreted as replacements for established thermodynamic correlations. Instead, they provide complementary, data-derived relationships that explicitly balance predictive performance, complexity, and physical plausibility under domain shift. The consistency between the symbolic expressions and the trends obtained from PDP and SHAP analysis further supports the interpretation that the discovered relationships capture stable physicochemical patterns rather than purely spurious correlations.

## 5 Conclusion

This study reframes drug solubility prediction in supercritical CO₂ as a domain-generalized, small-data, non-i.i.d. problem rather than a conventional regression task, where distributional shifts fundamentally challenge model reliability. The results establish that high predictive accuracy alone is insufficient, as models optimized for in-distribution performance fail to extrapolate reliably to unseen compounds. To address this, a unified framework was introduced that integrates symbolic regression with domain generalization techniques (IRM and DANN) within a multi-objective optimization setting. This framework enables the discovery of invariant, interpretable, and physically consistent equations, rather than relying on black-box predictions. Reliable solubility modeling requires moving beyond accuracy toward invariant, interpretable, and physically consistent representations. While GPR provides strong predictive performance and DANN improves robustness, symbolic regression plays a critical role by producing closed-form equations that align with thermodynamic principles and offer direct insight into extrapolation behavior. A key strength of the framework lies in its multi-level validation. The strong consistency observed across SHAP (local explanations), partial dependence plots (global behavior), and symbolic equations (analytical structure) confirms that the learned relationships are coherent and physically meaningful across domains. This agreement demonstrates that the model captures invariant structure rather than spurious correlations, which is essential for reliable generalization. Practically, the framework enables more reliable screening of unseen drug candidates, reduces experimental burden, and supports process design under uncertainty. Conceptually, it shifts machine learning from predictive fitting toward interpretable knowledge discovery. This work advances the field by establishing equation discovery under domain shift as a principled pathway toward transferable scientific understanding in complex physicochemical systems.

## Data Availability

The original contributions presented in the study are included in the article/Supplementary Material, further inquiries can be directed to the corresponding author.
